# 3-methylcrotonyl-CoA carboxylase deficiency: Clinical, biochemical, enzymatic and molecular studies in 88 individuals

**DOI:** 10.1186/1750-1172-7-31

**Published:** 2012-05-29

**Authors:** Sarah C Grünert, Martin Stucki, Raphael J Morscher, Terttu Suormala, Celine Bürer, Patricie Burda, Ernst Christensen, Can Ficicioglu, Jürgen Herwig, Stefan Kölker, Dorothea Möslinger, Elisabetta Pasquini, René Santer, K Otfried Schwab, Bridget Wilcken, Brian Fowler, Wyatt W Yue, Matthias R Baumgartner

**Affiliations:** 1 Division of Metabolism and Children’s Research Center (CRC), University Children’s Hospital Zurich, Steinwiesstraße 75, 8032, Zurich, Switzerland; 2 Center for Pediatrics and Adolescent Medicine, University Hospital Freiburg, Freiburg, Germany; 3 Zürich Center for Integrative Human Physiology (ZHIP), University of Zürich, Zürich, Switzerland; 4 Metabolic Unit, University Children's Hospital, Basel, Switzerland; 5 Department of Clinical Genetics, Rigshospitalet, Copenhagen, Denmark; 6 Children’s Hospital of Philadelphia, University of Pennsylvania, Perelman School of Medicine, Section of Biochemical Genetics, Philadelphia, Pennsylvania, USA; 7 University Children’s Hospital Frankfurt, Frankfurt, Germany; 8 Division of Inherited Metabolic Diseases, University Children's Hospital, Heidelberg, Germany; 9 Department of Pediatric and Adolescent Medicine, University Hospital Vienna, Vienna, Austria; 10 Metabolic and Muscular Unit, Clinic of Pediatric Neurology, Meyer Children's Hospital, Florence, Italy; 11 Department of Pediatrics, University Medical Center Hamburg-Eppendorf, Hamburg, Germany; 12 Department of Biochemical Genetics, The Children's Hospital at Westmead, Sydney, New South Wales, Australia; 13 Structural Genomics Consortium, University of Oxford, Oxford, UK

**Keywords:** 3-Methylcrotonyl-CoA carboxylase, MCCC1, MCCC2, Biotin, Inborn error, Organic aciduria, Newborn screening

## Abstract

**Background:**

Isolated 3-methylcrotonyl-CoA carboxylase (MCC) deficiency is an autosomal recessive disorder of leucine metabolism caused by mutations in *MCCC1* or *MCCC2* encoding the α and β subunit of MCC, respectively. The phenotype is highly variable ranging from acute neonatal onset with fatal outcome to asymptomatic adults.

**Methods:**

We report clinical, biochemical, enzymatic and mutation data of 88 MCC deficient individuals, 53 identified by newborn screening, 26 diagnosed due to clinical symptoms or positive family history and 9 mothers, identified following the positive newborn screening result of their baby.

**Results:**

Fifty-seven percent of patients were asymptomatic while 43% showed clinical symptoms, many of which were probably not related to MCC deficiency but due to ascertainment bias. However, 12 patients (5 of 53 identified by newborn screening) presented with acute metabolic decompensations. We identified 15 novel *MCCC1* and 16 novel *MCCC2* mutant alleles. Additionally, we report expression studies on 3 *MCCC1* and 8 *MCCC2* mutations and show an overview of all 132 *MCCC1* and *MCCC2* variants known to date.

**Conclusions:**

Our data confirm that MCC deficiency, despite low penetrance, may lead to a severe clinical phenotype resembling classical organic acidurias. However, neither the genotype nor the biochemical phenotype is helpful in predicting the clinical course.

## Background

Isolated 3-methylcrotonyl-CoA carboxylase (MCC) deficiency (MIM#s 210200 and 210210) is an autosomal recessive disorder of leucine metabolism [1]. The mitochondrial enzyme MCC (EC 6.4.1.4) catalyzes the fourth step in the leucine catabolic pathway and belongs to the family of biotin-dependent carboxylases, including acetyl-CoA carboxylase (ACC), propionyl-CoA carboxylase (PCC) and pyruvate carboxylase (PC) [1]. MCC consists of an alpha and a beta subunit assembled into a α_6_β_6_ dodecamer. The larger α subunit harbours the biotin carboxylase (BC) domain and the biotin carboxyl carrier protein domain covalently bound with a biotin prosthetic group, while the smaller β subunit contains the carboxyltransferase (CT) domain.

Isolated MCC deficiency is caused by mutations in the *MCCC1 (formerly MCCA)* or the *MCCC2 (formerly MCCB)* gene coding for the α and β subunit, respectively [2-4]. Human *MCCC1* has 19 exons and maps to chromosome region 3q25-q27, *MCCC2* consists of 17 exons and has been located to chromosome region 5q12-q13 [2-4]. A total of 49 *MCCC1* and 52 *MCCC2* mutations have been reported so far with the majority being missense mutations along with small insertions/deletions, nonsense, frameshift, and splice site mutations [2-12].

Increased urinary levels of 3-hydroxyisovaleric acid (3-HIVA) and 3-methylcrotonylglycine (3-MCG) are usually found in isolated MCC deficiency. Additionally, 3-hydroxyisovalerylcarnitine (C5OH) is characteristically present in blood and urine. Many patients also develop a severe secondary carnitine deficiency [1]. Surprisingly, MCC deficiency was found to be the most frequent organic aciduria detected in tandem mass spectrometry based newborn screening (NBS) programs in North America [13,14], Europe [15,16] and Australia [17].

The clinical picture of MCC deficiency is heterogeneous and often highly variable even within the same family [10,18]. The phenotype ranges from neonatal onset with severe neurological involvement and even lethal cases [19-21] to asymptomatic adults [3,6,11,22]. Some patients develop an acute metabolic crisis usually triggered by intercurrent infections or introduction of a protein-rich diet in early childhood. Symptoms include vomiting, opisthotonus, involuntary movements, seizures, coma and apnoea typically associated with metabolic acidosis, hypoglycemia and in some cases mild hyperammonemia [3,7,23-27]. Others present with neurological abnormalities such as seizures, muscular hypotonia or developmental delay [6,18,28-31].

In contrast, the majority of children diagnosed by NBS have been reported to have remained asymptomatic so far [6,7,11,32]. Moreover, several asymptomatic MCC-deficient mothers have been identified only by detection of abnormal metabolites in the neonatal-screening sample from their healthy babies [3,6,11,22] and a number of asymptomatic affected siblings have been identified by family screening [33-35]. The comparative analysis of published case reports with German NBS data indicated that probably less than 10% of affected individuals develop symptoms [6]. Therefore, MCC deficiency may be considered to be a genetic condition with low penetrance.

Therapeutic approaches comprise supplementation with oral L-carnitine and a diet modestly restricted in leucine but the efficacy of these approaches is unproven [36].

Here we summarize clinical, biochemical, enzymatic and molecular genetic data of 88 MCC-deficient individuals, present an update of all *MCCC1* and *MCCC2* mutations reported to date including 15 novel *MCCC1* and 16 novel *MCCC2* mutations and show expression studies of 10 missense mutations and one small deletion.

## Patients and methods

### Patients

Eighty-eight subjects with MCC deficiency from 78 families (10 sib-pairs) were included in this study (Tables 1, 2, 3 and 4). Cultured fibroblasts (n = 69) or genomic DNA (n = 16) from 85 individuals were sent to our laboratory for confirmation of MCC deficiency. In the remaining 3 subjects mutation analysis was performed in a genetic laboratory in the USA. Forty-five subjects were male, 40 were female; the sex of 3 individuals was not reported. Of the 88 individuals 45 (51%) were Caucasian, 27 (31%) Turkish, 8 (9%) Arab, 6 (7%) Asian, one (1%) African-American, and one patient (1%) was of mixed African Caucasian ancestry.

**Table 1:** Sociodemographic, biochemical, enzymatic, genetic and clinical information on 88 patients with MCC deficiency 53 Individuals identified by newborn screening without (n = 36) and with (n = 13) reported symptoms (n = 4 without clinical details)

**Pt #**	**Sex**	**Ethnic origin**	**Current age (y)**	**Biochemical phenotype**			**Carboxylase activities in fibroblasts (pmol/ min/mg protein)**^**1**^		**Genotype**			**Clinical phenotype**^**§**^
				**DBS/ plasma**	**urine**				**affected gene**	**Nucleotide change (at RNA level)**	**Amino acid change (predicted from RNA)**	
				**C5OH**	**3-HIVA**	**3MCG**	**MCC**	**PCC**		***Allele 1 Allele 2***		
20	f	Caucasian	10	++	++	++	15.4	812	*MCCC1*	c.1155A>C	p.R385S	asymptomatic (fr)
										c.559T>C	p.S187P	
21	f	Turkish	11	++	++	++	0	530	*MCCC2*	c.803G>C	p.R268T	asymptomatic (ltf, 0.3 y)
										(r.785_803del)	(p.G262_R268delfs*5)	
										c.803G>C	p.R268T	
										(r.785_803del)	(p.G262_R268delfs*5)	
22	f	Turkish	12	++	++	++	5.7	587	*MCCC2*	c.464G>A	p.R155Q	asymptomatic (fr)
										c.464G>A	p.R155Q	
23	f	Arab	12	++	++	++	0	594	*MCCC2*	c.469C>T	p.Q157*	asymptomatic (ltf, 1y)
										c.469C>T	p.Q157*	
25	m	Caucasian	11	++	++	++	na	na	*MCCC1*	c.872C>T◊ -	p.A291V -	asymptomatic (ltf)
26	f	Caucasian	9	++	++	++	na	na	*MCCC2*	c.1690T>C◊ -	p.X564QLE -	asymptomatic (fr)
27	f	Caucasian	10	++	++	++	1.1	305	*MCCC1*	c.1155A>C◊ -	R385S -	asymptomatic, but facial dysmorphies with hypertelorism, mongoloid palpebral fissures, low set ears, mild macroglossia, normal karyotype 46XX (ltf, 0.3 y)
29	m	Turkish	10	++	++	++	1.5	723	*MCCC2*	c.295G>C	p.E99Q	asymptomatic (ltf, 6y)
										c.1574+1G>A	(p.F497Gfs*4)	
34	m	Caucasian	9	++	++	++	16.2	542	*MCCC2*	c.845A>G	p.H282R	asymptomatic (fr)
										c.845A>G	p.H282R	
39	f	Caucasian	9	++	++	++	0.7	696	*MCCC2*	c.517dupT	p.S173Ffs*25	asymptomatic (fr)
										c.1123G>T	p.V375F	
40	m	Caucasian	9	++	++	++	5.1	620	*MCCC2*	c.214C>T	p.R72*	asymptomatic (fr)
										c.416_ 427del12ins16	p.T139_G143 >RWVPGEfs*35	
41	m	Caucasian	8	++	++	++	0	595	*MCCC1*	c.694C>T◊ -	p.R232W -	asymptomatic, mild developmental delay within the first years of life, normal development at present (fr)
43a	f	Caucasian	8	++	++	++	8.1	704	*MCCC1*	c.640_641delGG	p.G214Nfs*5	asymptomatic (ltf)
										c.1930G>T	p.E644*	
43b	f	Caucasian	7	++	++	++	na	na	*MCCC1*	c.640_641delGG	p.G214Nfs*5	asymptomatic (ltf)
										c.1930G>T	p.E644*	
52a	m	Turkish	9	+	+	++	1.6	603	*MCCC2*	c.803G>C	p.R268T	asymptomatic (ltf)
										(r.785_803del)	(p.G262_ R268delfs*5)	
										c.803G>C	p.R268T	
										(r.785_803del)	(p.G262_R268delfs*5)	
55	m	Asian	7	++	++	na	18.3	634	*MCCC2*	c.351_353delTGG	p.G118del	asymptomatic (ltf, 0.6y)
										c.659G>A	p.G220E	
56	m	Turkish	7	+	+	++	9.6	359	*MCCC2*	c.1567A>G	p.S523G	asymptomatic (fr)
										(exon 6 skipping)	(p.V171Dfs*20)	
57	m	Asian	7	++	++	++	10.2	541	*MCCC1*	c.863A>G	p.E288G	asymptomatic (fr)
										c.863A>G	p.E288G	
58	m	Turkish	7	++	++	++	5.2	1046	*MCCC2*	c.538C>T	p.R180*	asymptomatic (fr)
										c.538C>T	p.R180*	
62	m	Turkish	7	++	++	++	9.5	856	*MCCC1*	c.873+4524_ 6787del2264	p.?	asymptomatic (fr)
										c.873+4524_ 6787del2264	p.?	
64	f	Turkish	8	++	++	++	9.9	762	*MCCC2*	c.803G>C	p.R268T	asymptomatic (ltf, 5y)
										(r.785_803del)	(p.G262_R268delfs*5)	
										c.803G>C	p.R268T	
										(r.785_803del)	(p.G262_R268delfs*5)	
67	f	Turkish	10	++	na	na	48.4	1065	*MCCC2*	c.464G>A	p.R155Q	asymptomatic (fr)
										c.1015G>A	p.V339M	
70a	f	Caucasian	6	+	++	++	0	520	*MCCC1*	c.2079delA◊ -	p.V694* -	asymptomatic (fr)
72	m	Caucasian	7	++	++	++	11.5	451	*MCCC2*	c.455A>C	p.K152T	asymptomatic (ltf)
										c.903+6_ 903+9delTACG	p.?	
78	f	Caucasian	6	++	++	++	4.8	416	*MCCC2*	c.671C>T	p.P224L	asymptomatic (fr)
										c.671C>T	p.P224L	
82a	f	Caucasian	5	++	++	++	1.6	783	*MCCC2*	c.512-1G>A	p.?	asymptomatic (ltf, 1y)
										c.512-1G>A	p.?	
82b	f	Caucasian	5	++	++	++	na	na	*MCCC2*	c.512-1G>A	p.?	asymptomatic (ltf, 1y)
										c.512-1G>A	p.?	
91	m	Turkish	7	++	+	+	35.5	513	*MCCC2*	c.295G>C	p.E99Q	asymptomatic (fr)
										c.295G>C	p.E99Q	
93b	m	Caucasian	8	++	na	+	na	na	*na*	na	na	asymptomatic (ltf)
107	m	Caucasian	2	++	++	++	4.0	613	*MCCC2*	c.1073-12C>G		asymptomatic (fr)
										(r.1073_1216del+ r.1073insr.1073-48_ r.1073-1)	(p.G358Vfs*6+ p.G358Afs*12)	
										c.1073-12C>G		
										(r.1073_1216del+ r.1073insr.1073-48_ r.1073-1)	(p.G358Vfs*6+ p.G358Afs*12)	
112	m	Turkish	0.8	++	++	++	0	797	*MCCC2*	c.658_662delTCAGA c.658_662delTCAGA	p.S220Tfs*15 p.S220Tfs*15	asymptomatic, however hyperammonemia of 270 umol/l under leucine loading test (fr)
115	f	Caucasian	0.7	++	++	++	5.0	864	*MCCC1*	c.803C>A	p.A268D	asymptomatic (fr)
										c.1155A>C	p.R385S	
125	f	Arab	3	+	n	na	102	726	*MCCC2*	c.1423G>A	p. G475R	asymptomatic (fr)
										c.1423G>A	p. G475R	
126	m	Caucasian	3	+	+	na	60.6	791	*MCCC2*	c.1300G>C	p.V434L	asymptomatic (fr)
										c.1300G>C	p.V434L	
137	m	Caucasian	5	++	++	++	na	na	*MCCC2*	c.518C>T	p.S173L	asymptomatic (fr)
										c.518C>T	p.S173L	
138	m	Caucasian	1.5	++	++	++	na	na	*MCCC1*	c.1155A>C	p.R385S	asymptomatic (fr)
										c.2009_2043del35	p.A670Dfs*34	
24	f	Turkish	10	++	++	++	1.8	390	*MCCC2*	c.295G>C c.295G>C	p.E99Q p.E99Q	attention deficit hyperactivity disorder (fr)
28	m	Caucasian	10	++	++	++	1.1	318	*MCCC1*	c.1155A>C (exon 15 skipping)	p.R385S (p.V562*)	attention deficit hyperactivity disorder (fr)
46	m	Caucasian/ African American	9	++	++	++	20.0	1054	*MCCC1*	c.2088dupA c.1526delG	p.V697Sfs*19 p.C509Sfs*14	3 metabolic decompensations with vomiting, hypoglycaemia and ketonuria (ltf, 7.5y)
53	f	Caucasian	7	+	++	++	28.4	773	*MCCC1*	c.1155A>C c.1315G>A	R385S p.V439M	at the age of 6 months minor psychomotor delay (ltf)
59	m	Faroe Islands	7	++	++	++	7.4	1051	*MCCC1*	c.1526delG c.1526delG	p.C509Sfs*14 p.C509Sfs*14	muscular hypotonia, muscle wakness, impaired physical performance (fr)
71	f	Turkish	died at 5 weeks	++	++	++	1.7	597	*MCCC1*	c.1136G>A c.1136G>A	p.G379D p.G379D	metabolic crisis, floppy infant, myoclonic jerks, respiratory insufficiency requiring mechanical ventilation, deceased at age 6 weeks
74	m	African American	6	++	+	+	23.0	749	*MCCC1*	c.1302T>G c.2123dupA	p.I434M p.H708Qfs*8	several metabolic decompensations, mild speech delay, immunodeficiency due to CD 16 deficiency (fr)
81	f	Caucasian	5	+	+	n	21.5	233	*MCCC2*	c.1015G>A◊ -	p.V339M -	Trisomy 21, psychomotor retardation, muscular hypotonia (fr)
90	m	Turkish	7	+	+	+	23.8	483	*MCCC2*	c.295G>C c.1015G>A	p.E99Q p.V339M	truncal and perioral hypotonia (fr)
105	m	Caucasian	3	++	++	++	0	412	*MCCC1*	c.1155A>C c.1820delG	p.R385S p.S607Ifs*5	unpleasant odour, failure to thrive, several acute metabolic decompensations with mild hyperammonemia during infections (fr)
108	m	Asian	2.5	++	++	++	0.8	456	*MCCC2*	c.518C>T c.518C>T	p.S173L p.S173L	recurrent infections, muscular hypertonia and hyperreflexia in infancy (fr)
127	m	Arab	2	+	na	na	92,9	755	*MCCC2*	c.1423G>C	p.G475R	muscle weakness (fr)
										c.1423G>C	p.G475R	
136	f	Caucasian	8	++	++	++	na	na	*MCCC2*	c.1149+1G>T c.1149+1G>T	p.? p.?	3 metabolic decompensations with acidosis, hypoglycaemia, vomiting, encephalopathy and coma (fr)
31	f	Caucasian	10	na	na	na	12.4	518	*MCCC1*	c.1155A>C	p.R385S	?
										c.400G>A	p.E134K	
103	m	Caucasian	3	++	++	++	0	545	*MCCC2*	(exon 7 to 14 skipping)	(p.I209Pfs*43)	?
										(exon 7 to 14 skipping)	(p.I209Pfs*43)	
111	?	Caucasian	1	++	+	+	34.0	1083	*MCCC2*	c.1015G>A	p.V339M	?
										c.1309A>G		
										(r.1309A>G+ r.1310_1373del64)	(p.I437V+ p.I437Tfs*15)	
113	?	Caucasian	1	+	+	n	7.9	407	*MCCC1*	c.193A>T	p.M65L	?
										c.1193_1194delTG	p.V398Gfs*19	

^1^ control values measured in 53 cell lines, expressed as median value and (range): MCC activity, 305 pmol/min/mg protein (134-671); PCC activity, 583 (208-1165); ratio of PCC/MCC activity, 1.93 (1.19 – 2.58).

^§^information in brackets: fr followed regularly, ltf lost to follow-up, age of last follow-up, if known. + slightly elevated; ++ massively elevated; C5OH 3-hydroxyisovalerylcarnitine; DBS dried blood spots; 3-HIVA 3-hydroxyisovaleric acid; 3-MCG 3-methylcrotonylglycine; f female; fr followed regularly; ltf lost to follow-up; m male; MCC methylcrotonyl-CoA carboxylase; n normal; na not available; NBS newborn screening; PCC propionyl-CoA carboxylase; Pt # Patient number; RNA nd RNA not detectable; SMS selective metabolic screening; y years; ◊ mutation heterozygous on genomic PCR, homozygous in RT-PCR; # diagnosed following the positive NBS result of their baby; ? not known.

**Table 2:** Sociodemographic, biochemical, enzymatic, genetic and clinical information on 88 patients with MCC deficiency 18 individuals identified by selective metabolic screening due to clinical symptoms (n = 17, no clinical details in n = 1)

**Pt #**	**Sex**	**Ethnic origin**	**age at diagnosis**	**current age (y)**	**Biochemical phenotype**			**Carboxylase activities in fibroblasts (pmol/min/mg protein)**^**1**^		**Genotype**			**Clinical phenotype**^**§**^
					**DBS/ plasma**	**urine**				**affected gene**	**Nucleotide change (at RNA level)**	**Amino acid change (predicted from RNA)**	
					**C5OH**	**3-HIVA**	**3MCG**	**MCC**	**PCC**		***Allele 1 Allele 2***		
30	m	Turkish	newborn	died at 33 days	++	++	++	0	637	*MCCC2*	c.1574+1G>A c.1574+1G>A	(p.F497Gfs*4) (p.F497Gfs*4)	acute decompensation on first day of life, acidosis, hypoglycaemia, hyperlactemia, hyperammonemia, encephalopathy, depressed neonatal reflexes, hypertonic episodes, prominent hypotonia, respiratory insufficiency requiring assisted ventilation, cardiac arrest, patient deceased on day 33
													CT scan of the brain: multiple cysts, ventricular dilatation, cerebral atrophy
32a	m	Arab	4 years	14	++	++	++	5.0	863	*MCCC2*	c.127C>T c.127C>T	p.Q43* p.Q43*	muscular hypotonia, weakness, mild motor delay (fr)
35a	m	Caucasian	9 months	16	na	++	++	7.3	976	*MCCC2*	(exon 7 to 14 skipping) (exon 7 to 14 skipping)	(p.I209Pfs*43) (p.I209Pfs*43)	developmental delay, familial nystagmus, hyperopia, significant hand tremor, mild learning disability, failure to thrive, unpleasant odour descibed as "smelling like cat`s urine", hypothermia, ketonuria, hypoglycemia and mild hyperammonemia prior to stabilisation on dietary therapy (ltf, 3y)
36	f	Turkish	3 years	11	++	++	++	0.4	420	*MCCC1*	c.1527C>A c.1527C>A	p.C509* p.C509*	mental and speech retardation, spasticity, impaired physical performance (ltf)
42	f	Caucasian	?	24	++	++	++	0	664	*MCCC2*	c.929C>G c.929C>G	p.P310R p.P310R	severe muscular weakness, muscle pain (ltf, 16y)
44	m	Caucasian	1.5 years	10	na	++	++	4.0	425	*MCCC2*	c.463C>T c.463C>T	p.R155W p.R155W	psychomotor retardation, seizures, muscular hypotonia, metabolic stroke, failure to thrive, clinodactyly of the 5th fingers (fr)
50	f	Arab	13 years	21	na	++	++	8.1	761	*MCCC1*	c.1882G>T c.1114C>T	p.E628* p.Q372*	mild Reye-like episode and encephalitis during Influenza A infection at age 5 years, mild learning disability, severe attention-deficit hyperactivity disorder, multiple sclerosis (fr)
54	m	Asian	?	13	++	++	++	1.3	1162	*MCCC1*	c.980C>G c.639+2T>A	p.S327* p.S164Rfs*3	psychomotor retardation, attention deficit hyperactivity disorder, frequent skin picking behaviour (ltf)
60	f	Turkish	?	10	++	++	++	6.4	754	*MCCC1*	c.2079delA c.2079delA	p.V694* p.V694*	mild global psychomotor retardation, convulsions starting at the age of 18 months during febrile episode, continued as generalized tonic clonic seizures after the age of 3 years, nephrolithiasis, episodes of hematuria (ltf, 4 y)
63	m	Turkish	?	8	++	++	na	12.0	729	*MCCC2*	c.464G>A c.464G>A	p.R155Q p.R155Q	3 metabolic decompensations with encephalopathy, seizures, acidosis, hypoglycemia, mild developmental retardation
68	m	Turkish	3 years	9	++	++	++	2.4	335	*MCCC1*	c.1155A>C c.1155A>C	R385S R385S	severe metabolic decompensation with metabolic stroke, cerebral edema and hemiparesis, mild psychomotor retardation, seizures (fr)
77	m	Arab	8 months	9	na	++	++	0	777	*MCCC2*	c.463C>T c.463C>T	p.R155W p.R155W	psychomotor and speech retardation, kyphoscolisis, genu varum, hypogammaglobulinemia, chronic diarrhea, reversible cytopenia under TPN (ltf, 7y)
80	m	Turkish	1.5 years	9	++	++	n (6m)++ (1y)	22.8	1162	*MCCC2*	c.116C>T c.116C>T	p.S39F p.S39F	speech retardation, seizures, recurring attacks of status epilepticus (ltf, 3y)
89	f	Caucasian	7 months	10	na	na	na	17.0	986	*MCCC2*	(exon 8 to 10 skipping)	(p.K248_V334del)	failure to thrive, poor feeding (ltf, 5y)
											(exon 8 to 10 skipping)	(p.K248_V334del)	
92	m	Caucasian	1 week	5	++	++	++	na	na	*MCCC2*	c.710G>A c.1149+5G>C	p.G237D p.?	acute metabolic crisis, mild retardation (fr)
96a	m	Turkish	1 year	6	++	++	++	7.3	1212	*MCCC1*	c.873+ 4524_6787del2264	large deletion	acidosis at 1 year of age, atonic seizures starting at 1 year of age (fr)
											c.873+ 4524_6787del2264	large deletion	
99a	f	Turkish	8 years	died at 8 years	++	++	++	na	na	*MCCC2*	c.392G>T c.392G>T	p.C131F p.C131F	catecholaminergic ventricular tachycardia (mutation in RyR2 gene) sudden cardiac death at age 8 years
69	?	Arab	?	9	na	na	na	18.9	1210	*MCCC2*	c.1567A>G	p.S523G	?
											c.1567A>G	p.S523G	

^1^ control values measured in 53 cell lines, expressed as median value and (range): MCC activity, 305 pmol/min/mg protein (134-671); PCC activity, 583 (208-1165); ratio of PCC/MCC activity, 1.93 (1.19 – 2.58).

^§^ information in brackets: fr followed regularly, ltf lost to follow-up, age of last follow-up, if known. + slightly elevated; ++ massively elevated; C5OH 3-hydroxyisovalerylcarnitine; DBS dried blood spots; 3-HIVA 3-hydroxyisovaleric acid; 3-MCG 3-methylcrotonylglycine; f female; fr followed regularly; ltf lost to follow-up; m male; MCC methylcrotonyl-CoA carboxylase; n normal; na not available; NBS newborn screening; PCC propionyl-CoA carboxylase; Pt # Patient number; RNA nd RNA not detectable; SMS selective metabolic screening; y years; ◊ mutation heterozygous on genomic PCR, homozygous in RT-PCR; # diagnosed following the positive NBS result of their baby; ? not known.

**Table 3:** Sociodemographic, biochemical, enzymatic, genetic and clinical information on 88 patients with MCC deficiency 8 individuals identified by family screening (asymptomatic individuals (n = 3), symptomatic individuals (n = 3), no clinical data (n = 2))

**Pt #**	**Sex**	**Ethnic origin**	**Age at diagnosis**	**Current age (y)**	**Biochemical phenotype**			**Carboxylase activities in fibroblasts (pmol/min/ mg protein)**^**1**^		**Genotype**			**Clinical phenotype**^**§**^
					**DBS/ plasma**	**urine**				**affected gene**	**Nucleotide change (at RNA level)**	**Amino acid change (predicted from RNA)**	
					**C5OH**	**3-HIVA**	**3MCG**	**MCC**	**PCC**		***Allele 1 Allele 2***		
32b	m	Arab	17 years	28	++	++	++	5.3	409	*na*	na	na	asymptomatic (fr)
93a	m	Caucasian	4 years	12	++	+	+	19.0	402	*MCCC1*	c.558delA	p.Q186Hfs*6	asymptomatic (ltf)
											c.558delA	p.Q186Hfs*6	
99b	f	Turkish	5.5 years	8	++	++	++	na	na	*na*	na	na	asymptomatic (fr)
70b	m	Caucasian	3.5 years	10	+	+	++	na	na	*na*	na	na	speech retardation, muscle weakness, hyperactivity, refusal of meat (fr)
96c	m	Turkish	3 years	8	++	++	++	na	na	*MCCC1*	c.873+4524_ 6787del2264	large deletion	mild speech retardation, macrocephaly (ltf)
											c.873+4524_ 6787del2264	large deletion	
35b	f	Caucasian	18 months	18	na	++	++	na	na	*na*	na	na	psychomotor retardation (by 2 years developmental age of 10 months), failure to thrive, hypothermia and ketonuria prior to stabilisation on dietary therapy (ltf, 1.75y)
52b	m	Turkish	?	?	na	na	na	na	na	*MCCC2*	c.803G>C	p.R268T	?
											(r.785_803del)	(p.G262_ R268delfs*5)	
											c.803G>C	p.R268T	
											(r.785_803del)	(p.G262_ R268delfs*5)	
52c	m	Turkish	?	?	na	na	na	na	na	*MCCC2*	c.803G>C	p.R268T	?
											(r.785_803del)	(p.G262_ R268delfs*5)	
											c.803G>C	p.R268T	
											(r.785_803del)	(p.G262_ R268delfs*5)	

^1^ control values measured in 53 cell lines, expressed as median value and (range): MCC activity, 305 pmol/min/mg protein (134-671); PCC activity, 583 (208-1165); ratio of PCC/MCC activity, 1.93 (1.19 – 2.58).

^§^information in brackets: fr followed regularly, ltf lost to follow-up, age of last follow-up, if known. + slightly elevated; ++ massively elevated; C5OH 3-hydroxyisovalerylcarnitine; DBS dried blood spots; 3-HIVA 3-hydroxyisovaleric acid; 3-MCG 3-methylcrotonylglycine; f female; fr followed regularly; ltf lost to follow-up; m male; MCC methylcrotonyl-CoA carboxylase; n normal; na not available; NBS newborn screening; PCC propionyl-CoA carboxylase; Pt # Patient number; RNA nd RNA not detectable; SMS selective metabolic screening; y years; ◊ mutation heterozygous on genomic PCR, homozygous in RT-PCR; # diagnosed following the positive NBS result of their baby; ? not known.

**Table 4:** Sociodemographic, biochemical, enzymatic, genetic and clinical information on 88 patients with MCC deficiency Mothers identified following the positive newborn screening result of their offspring (n = 9)

**Pt #**	**Sex**	**Ethnic origin**	**Age at diagnosis**	**Current age (y)**	**Biochemical phenotype**			**Carboxylase activities in fibroblasts (pmol/min/mg protein)**^**1**^		**Genotype**			**Clinical phenotype**^**§**^
					**DBS/ plasma**	**urine**				**affected gene**	**Nucleotide change (at RNA level)**	**Amino acid change (predicted from RNA)**	
					**C5OH**	**3-HIVA**	**3MCG**	**MCC**	**PCC**		***Allele 1 Allele 2***		
37	f	Asian	32 years	40	++	++	++	9.6	1268	*MCCC2*	c.1367C>T	p.A456V	asymptomatic (ltf)
											c.1367C>T	p.A456V	
51	f	Asian	24 years	32	++	na	na	0	475	*MCCC2*	c.351_353delTGG◊ -	p.G118del -	asymptomatic (ltf)
73c	f	Faroe Islands	29 years	37	++	++	++	na	na	*MCCC1*	c.1526delG	p.C509Sfs*14	asymptomatic (fr)
											c.1526delG	p.C509Sfs*14	
83	f	Caucasian	?	38	++	++	++	na	na	*MCCC1*	c.539G>T	p.G180V	asymptomatic (fr)
											c.558delA	p.Q186Hfs*6	
85	f	Caucasian	38 years	49	++	+	n	na	na	*MCCC2*	c.517dupT	p.S173Ffs*25	asymptomatic (ltf)
											c.599T>A	p.I200N	
100	f	Caucasian	29 years	34	++	++	++	na	na	*MCCC2*	c.505T>G	p.Y169D	asymptomatic (fr)
											c.1073-12C>G		
											(r.1073_1216del+ r.1073insr.1073- 48_r.1073-1)	(p.G358Vfs*6+ p.G358Afs*12)	
66	f	Caucasian	34 years	41	+	+	++	10.0	807	*MCCC2*	c.436T>Ac.416_427del12ins16	p.Y146Np.T139_G143> RWVPGEfs*35	several metabolic crises with hypoglycemia during febrile illnesses, metabolic stroke, cardiomopathy, paraesthesias (ltf)
87	f	Faroe Islands	28 years	33	++	n	n	13.0	826	*MCCC1*	c.1526delGc.1526delG	p.C509Sfs*14p.C509Sfs*14	chronic tiredness (fr), otherwise asymptomatic
33	f	Turkish	36 years	45	++	++	++	4.6	520	*MCCC2*	c.282-1G>C	p.S95_G128del	?
											c.282-1G>C	p.S95_G128del	

^1^ control values measured in 53 cell lines, expressed as median value and (range): MCC activity, 305 pmol/min/mg protein (134-671); PCC activity, 583 (208-1165); ratio of PCC/MCC activity, 1.93 (1.19 – 2.58).

§ information in brackets: fr followed regularly, ltf lost to follow-up, age of last follow-up, if known.

+ slightly elevated.

++ massively elevated; C5OH 3-hydroxyisovalerylcarnitine; DBS dried blood spots; 3-HIVA 3-hydroxyisovaleric acid; 3-MCG 3-methylcrotonylglycine; f female; fr followed regularly; ltf lost to follow-up; m male; MCC methylcrotonyl-CoA carboxylase

n normal; na not available; NBS newborn screening; PCC propionyl-CoA carboxylase; Pt # Patient number; RNA nd RNA not detectable; SMS selective metabolic screening; y years

^◊^ mutation heterozygous on genomic PCR, homozygous in RT-PCR; ^#^ diagnosed following the positive NBS result of their baby; ? not known.

Clinical, biochemical, enzymatic or mutation data of 32 individuals have been reported earlier [proband 20-32a, 33-34, 36-43a, 44 and 46 [11], 30 [23], 35a and 35b [30], 42 [37], 44 [38], 50 [28], 80 [9], 81 [39], 96a and 96c [10], and 136 [26]].

### Clinical data

A questionnaire was designed and sent out to the treating physicians. This questionnaire specifically addressed the mode of diagnosis, clinical symptoms, the psychomotor development, biochemical markers and long-term treatment regimens. Additionally, in some cases medical reports that were sent in together with the diagnostic samples were available and used for clinical data collection.

### Cell lines and enzyme assays

Fibroblasts were cultured in a culture medium containing 10% foetal calf serum, and the activities of PCC and MCC were assayed simultaneously in crude fibroblast homogenates by measuring the incorporation of ^14^C-bicarbonate into acid-non-volatile products as described earlier [40].

### MCCC1 and MCCC2 mutation analysis by RT-PCR and genomic PCR

After obtaining informed consent mutation analysis was performed in 83 individuals. The 5 individuals in whom no mutation analysis was performed were siblings of index cases and presented with a metabolite profile typical for MCC deficiency. In probands from whom RNA and DNA were available RT-PCR amplification and sequencing of the entire *MCCC2* ORF was first performed and if no clearly pathogenic coding alterations were detected in *MCCC2,* the entire *MCCC1* ORF was also analyzed. Identified mutations were confirmed by PCR amplification of genomic DNA. In individuals from whom only DNA was available amplification of all *MCCC1* and *MCCC2* exons and flanking intronic sequences from genomic DNA followed by direct sequencing was performed.

RNA and genomic DNA were extracted from cultured skin fibroblasts or peripheral blood leukocytes using the QIAamp® RNeasy or QIAamp® RNA Blood Mini Kit and the QIAamp® DNA Mini Kit (Qiagen AG, Basel, Switzerland), respectively. The RT-PCR reaction was performed using the One-Step RT-PCR kit (Qiagen AG, Basel, Switzerland) following the manufacturer’s instructions. First-strand *MCCC1* and *MCCC2* cDNA was amplified as described [2]. PCR products were sequenced in a thermocycler and analyzed with an ABI Prism 3100 Avant using the dye-terminator method (Applied Biosystem, Rotkreuz, Switzerland) according to the manufacturer’s instructions. To confirm mutations identified in RT-PCR products, a genomic fragment containing the corresponding exon was amplified using flanking intronic primers, and the PCR product was sequenced directly. In cases where only one of the two alleles could be identified in the standard RT-PCR product all exons and flanking intronic regions were sequenced. The sequences of all primers are available upon request.

To exclude that the identified missense mutations are common polymorphisms we amplified the relevant exons from genomic DNA of 100 controls (200 alleles).

### Construction of wild type and mutant MCCC1 and MCCC2 expression vectors and transfections

The following mutations were introduced by site-directed mutagenesis into the existing wildtype MCCC1 and MCCC2 pCR Blunt II TOPO vector (Invitrogen, Basel, Switzerland): *MCCC1* p.E288G, p.G379D, p.I434M and *MCCC2* p.S39F, p.G118del, p.Y146N, p.H282R, p.V434L, p.A456V, p.G475R, p.S523G. All constructs were then transferred into the mammalian expression vector pTracer-CMV2 (Invitrogen, Basel, Switzerland) and seq-uenced for validation. For expression studies the constructs were transiently transfected into transformed cultured fibroblasts deficient in either MCCC1 (homozygous for c.1264_1265insG/p.Q422Rfs*10 or compound heterozygous for c.1264_1265insG/p.Q422Rfs*10 and c.1682-3A > G/p.N561Kfs*10) or MCCC2 (homozygous for c.127 C > T/p.Q43*) by electroporation as described [2]. The cells were harvested 48 hours later and assayed for MCC and PCC activity.

### Western blot analysis of expressed proteins

Western blot analysis of proteins extracted from cells harvested 48 h after transfection was performed as described earlier [41]. For immunostaining of MCCC2 a commercially available antibody was used (Abnova). Antiserum for MCCC1 was produced by inoculating rabbits with peptides corresponding to the hydrophilic strech of the last 19 C-terminal amino acids (RHTPLVEFEEEESDKRESE) of human MCCC1 conjugated to keyhole limpet hemocyanin (Covance, Denver, Colorado). β-Actin was stained as control.

The biotin-containing MCC and PCC α-subunits and PC were also stained using streptavidin-alkaline phosphatase followed by colorimetric detection (Transcend™ Non-Radioactive Translation Detection Systems Kit, Promega, Dübendorf, Switzerland).

### In silico prediction of functional relevance of identified mutations

The human MCCC1/2 enzyme has not been structurally characterized. Missense mutations were therefore interpreted structurally using the homologous structure of *Pseudomonas aeruginosa* MCC holoenzyme (PDB code 3U9T). Amino acid sequence alignment of MCCC1/2, ACC1/2, PCCα/β sequences was constructed using the ICM-Pro program (Molsoft, San Diego) with the implemented alignment algorithm. The protein sequences NG_008100.1 and NG_008882.1 [GenBank at the NCBI] were used as reference sequences for the alpha and beta subunit of MCC, respectively.

## Results

A comprehensive summary of clinical, biochemical, enzymatic and molecular genetic information on each patient is given in Tables 1, 2, 3 and 4.

Diagnosis of MCC deficiency was confirmed by assaying MCC and PCC activities in fibroblasts of 68 individuals. In 50 cell lines MCC activity was severely reduced to less than 5% of the median control value. In 16 further cell lines residual MCC activity varied between 5.1% and 20% and in 2 cell lines MCC activity was 31% and 34% of the control value. All cell lines had a highly increased PCC/MCC activity ratio of at least 7.1. From 20 subjects fibroblasts were not available and the diagnosis was confirmed by mutation analysis using genomic DNA.

### Clinical data

Twenty-six individuals (30%) were diagnosed by selective metabolic screening (SMS) due to clinical symptoms (n = 18) or a positive family history (n = 8) while 53 individuals (60%) were identified by expanded NBS. Additionally, 9 mothers (10%) were diagnosed following a positive NBS result of their healthy offspring. In patients in whom a metabolic work-up was initiated due to clinical symptoms age at diagnosis ranged between one week and 13 years (median 1.5 years) (Table 2). Patients identified by family screening had a median age at diagnosis of 3.75 years (range 1.5-17 years) (Table 3), and mothers were diagnosed at a median age of 30.5 years (range 24-38 years) (Table 4).

Clinical information was available from 80 individuals. Parental consanguinity was reported in 31 subjects with most parents being second-degree relatives. Fourty-four parents were non-consanguineous and of 13 individuals no information on consanguinity was available. Three children had deceased, two during an acute metabolic decompensation at the age of 33 days and 6 weeks, and the third, a 8-year-old girl with sudden cardiac death due to catecholaminergic polymorphic ventricular tachycardia (mutations in *RyR2* gene).

**Figure 1:**
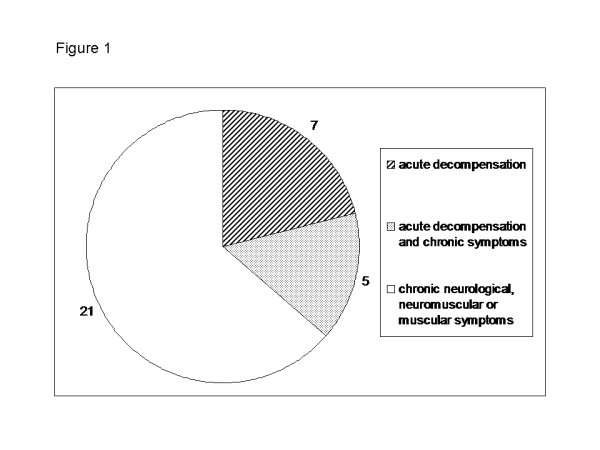
**Clinical manifestation of 33 symptomatic individuals with MCC deficiency.** One patient who died of sudden cardiac arrest at the age of 8 years was excluded from this figure as catecholaminergic ventricular tachycardia with mutations in the *RyR2* gene was identified as a likely cause for the cardiac symptoms.

In 34 (43%) of 80 subjects clinical symptoms were reported ranging from acute metabolic decompensation with ketoacidosis, hypoglycemia and encephalopathy to neuromuscular symptoms, mental retardation or attention deficit hyperactivity disorders (Figure 1). Twelve patients, 5 of which were diagnosed by newborn screening, had at least one acute metabolic decompensation. The most common clinical symptoms of acute crises were vomiting and encephalopathy with impaired conciousness. Neurologic symptoms like seizures, metabolic stroke, hemiparesis and cerebral edema were less frequent. The most common laboratory findings were acidosis and hypoglycaemia. Among chronic symptoms mental retardation including speech retardation were the most common findings followed by seizures, muscular hypotonia, muscle weakness, muscle pain and failure to thrive. In 5 patients an attention deficit hyperactivity disorder was reported.

Thirty-five out of 61 (57%) living individuals of whom recent follow-up information was available or who had been followed for at least until the age of three years have remained asymptomatic. Notably, 25 (69%) of the 36 subjects identified by NBS of whom either recent information or follow-up data until the age of at least three years were available, have remained without symptoms.

Sixty-nine out of 75 individuals of whom information on treatment was available received dietary and/or medical therapy. Forty individuals were given a protein-restricted or leucine-restricted diet (which was stopped later in life in some cases) and in 23 a leucine-free amino acid mixture was administered at least temporarily. In 30 subjects oral biotin was given on a trial basis. 63 patients were supplemented with oral carnitine, and 10 patients received oral glycine.

### Biochemical phenotype at diagnosis

Presence of C5OH in blood or dried blood spots and presence of elevated urinary excretion of 3-HIVA and 3-MCG at the time of diagnosis are shown in Tables 1, 2, 3 and 4. A biochemical phenotype characteristic for MCC deficiency with elevated excretion of 3-HIVA and/or 3-MCG, defined as more than twice the upper normal value, was found in 85% (68/80) of individuals. In 14% (11/80) only mildly elevated excretion was detected. In one patient (1%) no elevated excretion of 3-HIVA and 3-MCG was detected. Notably, 5 individuals including one patient reported earlier [39] did not excrete 3-MCG, the pathognomonic metabolite of MCC deficiency. However, one of these individuals showed massive excretion of 3-MCG when re-evaluated 6 months later.

C5OH in dried blood spots was highly elevated in 85% (66/78) and slightly (less than twice the upper normal value) elevated in 15% (12/78) of individuals. Low C5OH concentrations were not always linked with low urinary excretion of metabolites and vice versa.

In 43/68 individuals (63%) a secondary carnitine deficiency was present. Remarkably, 24 (60%) of those 40 children identified through NBS of whom information on free carnitine concentrations was available had decreased free carnitine levels already at the time of diagnosis or within the neonatal period. In 15 of them free carnitine concentration was below 5 μmol/l.

### Mutation analysis

Of the 83 individuals in whom mutation analysis was performed, 31 had mutations in *MCCC1* and 52 in *MCCC2* (Tables 1, 2, 3 and 4). Forty-eight probands were found to be homozygous (12 for *MCCC1* mutations and 36 for *MCCC2* mutations) while 28 were compound heterozygous (15 for *MCCC1* mutations and 13 for *MCCC2* mutations). In 5 of these patients RT-PCR results showed exon skipping either for one or both alleles, but the underlying genomic mutation could not be identified. In the remaining 7 subjects (4 *MCCC1* and 3 *MCCC2*) it was not possible to detect a second mutation in spite of sequencing all exons and flanking intronic sequences. However, the mutant allele identified appeared to be homozygous in RT-PCR, but was clearly heterozygous in genomic DNA. This suggests that the steady state level of mRNA from the second allele was not detectable as would be the case for a promoter mutation or an intragenic deletion or insertion missed by genomic PCR.

We identified a total of 15 novel *MCCC1* and 16 novel *MCCC2* mutations (shown in bold in Tables 5 and 6). The 15 novel *MCCC1* mutations comprise 7 missense, 2 nonsense, 1 splice site and 5 frameshift mutations (5 due to small deletions and one due to a small insertion). The 16 novel *MCCC2* variants include 11 missense and 4 splice site mutations and 1 deletion of a single amino acid.

**Table 5:** **Overview on 64*MCCC1*mutant alleles and their consequences**

**Exon/Intron**	**Nucleotide change at cDNA level**	**Amino acid change (at RNA level)**	**Consequence**	**Patients, in whom this mutation was found in this study/ Reference of first description of the mutation**
exon 1	c.43GC>T	p.E15*	nonsense	Morscher et al. 2012
intron 1	c.89+2_89+34del	p.?	splice	Morscher et al. 2012
intron 2	c.137-2A>G	p.?	splice	Stadler et al. 2006
exon 3	c.137G>A	p.G46E	missense	Nguyen et al. 2011
exon 3	c.168C>G	p.N56K	missense	Morscher et al. 2012
**exon 3**	**c.193A>T**	**p.M65L**	**missense**	**#113/ This study**
exon 3	c.227_228delTG	p.V76Gfs*4	frameshift	unpublished^a^
exon 3	c.251_252delGA^b^	p.R84Kfs*10	deletion/frameshift	Stadler et al. 2006
exon 4	c.369G>C	p.Q123H	missense	Stadler et al. 2006
exon 5	c.375C>G	p.I125M	missense	Stadler et al. 2006
exon 5	c.400G>A	p.E134K	missense	#32/ Dantas et al. 2005
exon 5	c.479T>G	p.M160R	missense	Stadler et al. 2006
**exon 6**	**c.539G>T**	**p.G180V**	**missense**	**#83/ This study**
exon 6	c.558delA	p.Q186Hfs*6	deletion/frameshift	#83, 93a/ Morscher et al. 2012
exon 6	c.559T>C	p.S187P	missense	#20, Dantas et al. 2005
**intron 6**	**c.639+2T>A**	p.S164Rfs*3	**splice, exon 6 skipping**	**#54/ This study**
exon 7	c.640_641delGG	p.G214Nfs*5^c^	deletion/frameshift	#43a, 43b/ Dantas et al. 2005
**exon 7**	**c.658_662delTCAGA**	p.S220Tfs*15	**deletion/frameshift**	**#112/ This study**
exon 7	c.694C>T	p.R232W	missense	#41/ Dantas et al. 2005
intron 7	c.762-1G>A	p. ?	splice	Nguyen et al. 2011
**exon 8**	**c.803C>A**	**p.A268D**	**missense**	**#115/ This study**
exon 8	c.841C>T	p.R281*	nonsense	Morscher et al. 2012
exon 8	c.842G>A	p.R281Q	missense	Morscher et al. 2012
**exon 8**	**c.863A>G**	**p.E288G**	**missense**	**#57/ This study**
exon 8	c.866C>T	p.A289V	missense	Baumgartner et al. 2001
exon 8	c.872C>T	p.A291V	missense	#25/ Dantas et al. 2005
intron 8 + exon 9	c.873+4524_6787del2264	2 transkripts: p.P292Gfs*18 p.P292_R361del	large deletion, **exon 9 and exon 9 and 10 skipping**	#62, 96a, 96c/ Eminoglu et al. 2009
exon 9	c.901_902delAA	p.K301Afs*10	deletion/ frameshift	Uematsu et al. 2007
exon 9	c.945T>A	p.Y315*	nonsense	Stadler et al. 2006
exon 10	c.974T>G	p.M325R	missense	Gallardo et al. 2001
exon 10	c.980C>G	p.S327*	nonsense	#54/ Morscher et al. 2012
exon 10	not published	p.Q372P	missense	Desviat et al. 2003
**exon 11**	**c.1114C>T**	p.Q372*	**nonsense**	**#50/ This study**
exon 11	c.1135G>A	p.G379S	missense	Stadler et al. 2006
**exon 11**	**c.1136G>A**	**p.G379D**	**missense**	**#71/ This study**
exon 11	c.1139A>C	p.H380P	missense	Morscher et al. 2012
exon 11	c.1155A>C	p.R385S	missense	#20, 27, 28, 31, 53, 68, 105, 115, 138/ Baumgartner et al. 2001, Gallardo et al. 2001
**exon 11**	**c.1193_1194delTG**	p.V398Gfs*19	**deletion/frameshift**	**#113/ This study**
exon 11	c.1225C>T	p.R409*	nonsense	Stadler et al. 2006
exon 11	c.1264_1265insG^d^	p.Q422Rfs*10^d^	insertion/frameshift	Baumgartner et al. 2001
intron 11	c.1268-2A>G	p.G423Efs*15	splice, exon 12/13 skipping	Stadler et al. 2006
**exon 12**	**c.1302T>G**	**p.I434M**	**missense**	**#74/ This study**
exon 12	c.1310T>C	p.L437P	missense	Baumgartner et al. 2001
**exon 12**	**c.1315G>A**	**p.V439M**	**missense**	**#53/ This study**
exon 12	c.1333C>T	p.Q445*	nonsense	Morscher et al. 2011
exon13	c.1380T>G	p.I460M	missense	Uematsu et al. 2007
exon 13	c.1522_1544del	p.L508Hfs*17	deletion	Morscher et al. 2012
exon 13	c.1526delG^e^	p.C509Sfs*14	deletion/frameshift	#46, 59, 73c, 87/ Dantas et al. 2005
exon 13	c.1527C>A	p.C509*	nonsense	#36/ Dantas et al. 2005
exon 13	c.1541dupG	p.L515Sfs*18	insertion/frameshift	Morscher et al. 2012
exon 13	c.1594G>C	p.D532H	splice	Baumgartner et al. 2001
intron 13	c.1594+3A>G	p.V461Nfs*13	splice, exon 13 skipping	Morscher et al. 2012
exon 14	c.1604C>T	p.S535F	missense	Holzinger et al. 2001
intron 14	c.1681+5G>A	p.Q533_N561del	splice, exon 14 skipping	Stadler et al. 2006
intron 14	c.1682-3A>G	p.N561Kfs*10	splice/frameshift	Dantas et al. 2005
exon 15	c.1695_1700del	p.V566_T567del	deletion	Morscher et al. 2012
exon 16	c.1750C>T	p.Q584*	nonsense	Uematsu et al. 2007
**exon 16**	**c.1820delG**	p.S607Ifs*5	**deletion/frameshift**	**#103/ This study**
**exon 17**	**c.1882G>T**	p.E628*	**nonsense**	**#50/ This study**
exon 17	c.1930G>T	p.E644*	nonsense	#43a, 43b/ Dantas et al. 2005
**exon 18**	**c.2009_2043del35**	p.A670Dfs*34	**deletion/frameshift**	**#138/ This study**
exon 19	c.2079delA	p.V694*	nonsense	#60, 70a/ Holzinger et al. 2001
exon 19	c.2088dupA	p.V697Sfs*19	insertion/frameshift	#46/ Dantas et al. 2005
**exon 19**	**c.2123dupA**	p.H708Qfs*8	**insertion/frameshift**	**#74/ This study**

^a^ Found in our laboratory in a heterozygous individual, not yet published.^b^ Published in the original paper as c.250_251delAG (p.R84Kfs*9), however AG is not found at this position in the reference sequence, but GA instead.^c^ Published in the original paper as p.G214IfsX5, nomenclature has been adapted to new approved guidelines.^d^ Published in the original paper as c.1264insG, Q421fs(+1), nomenclature has been adapted to new approved guidelines.^e^ Mainly found in patients from the Faroe Islands.

(NG_008100.1 [GenBank at the NCBI] was used as reference sequence. Consensus nomenclature according to approved guidelines ( http://www.hgvs.org/mutnomen/))

**Table 6:** **Overview on 68*MCCC2*mutant alleles and their consequences**

**Exon/Intron**	**Nucleotide change at cDNA level**	**Amino acid change (at RNA level)**	**Consequence**	**Patients, in whom this mutation was found in this study/ Reference of first description of the mutation**
exon 1	c.116C>T	p.S39F	missense	#80/ Dirik et al. 2008
exon 1	c.127C>T	p.Q43*	nonsense	#32/ Dantas et al. 2005
exon 3	c.214C>T	p.R72*	nonsense	#40/ Dantas et al. 2005
exon 3	c.243dupT	p.L81Ifs*7^a^	insertion/frameshift	Stadler et al. 2006
intron 3	c.281+5G>A	p.?	splice	Stadler et al. 2006
intron 3	c.281+5G>T	p.G67Lfs*35^b^	splice/exon 3 skipping^b^	Gallardo et al. 2001
intron3	c.282-1G>C	p.S95_G128del^c^	splice/exon 4 skipping	#33/ Dantas et al. 2005
exon 4	c.295G>C	p.E99Q	missense	#24, 29, 90, 91/ Baumgartner et al. 2001, Holzinger et al. 2001
exon 4	c.302C>T	p.S101F	missense	Stadler et al. 2006
**exon 4**	**c.351_353delTGG**	**p.G118del**	**deletion**	**#51, 55/ This study**
intron 4	c.383+1G>T	p.?	splice	Stadler et al. 2006
intron 4	c.384-2A>G	p.?	splice	Stadler et al. 2006
**exon 5**	**c.392G>T**	**p.C131F**	**missense**	**#99a/ This study**
exon 5	c.416_427del12ins16	p.T139_G143>RWVPGEfs*35	deletion/insertion/frameshift	#40, 66/ Dantas et al. 2005
**exon 5**	**c.436T>A**	**p.Y146N**	**missense**	**#66/ This study**
**exon 5**	**c.455A>C**	**p.K152T**	**missense**	**#72/ This study**
exon 5	c.463C>T	p.R155W	missense	#44, 77/ Dantas et al. 2005
exon 5	c.464G>A	p.R155Q	missense	#22, 63, 67/ Baumgartner et al. 2001
exon 5	c.469C>T	p.Q157*	nonsense	#23/ Dantas et al. 2005
exon 5	c.499T>C	p.C167R	missense	Gallardo et al. 2001
**exon 5**	**c.505T>G**	**p.Y169D**	**missense**	**#100/ This study**
intron 5	c.512-1G>A^d^	p.?	splice	#82a, 82b/ Baumgartner et al. 2001
exon 6	c.517dupT	p.S173Ffs*25	insertion/frameshift	#39, 85/ Baumgartner et al. 2001,Gallardo et al 2001
exon 6	c.518C>T	p.S173L	missense	#108, 137/ Baumgartner et al. 2001
exon 6	c.538C>T	p.R180*	nonsense	#58/ Stadler et al. 2006
exon 6	c.568C>T	p.H190Y	missense	Dantas et al. 2005
exon 6	c.569A>G	p.H190R	missense	Uematsu et al. 2007
exon 6	c.577C>T	p.R193C	missense	Baumgartner et al. 2001
exon 6	c.578G>A	p.R193H	missense	Stadler et al. 2006
exon 6	c.592C>T	p.Q198*	nonsense	Uematsu et al. 2007
**exon 6**	**c.599T>A**	**p.I200N**	**missense**	**#85/ This study**
exon 7	c.652G>A	p.A218T	missense	Gallardo et al. 2001
exon 7	c.653C>T	p.A218V	missense	Morscher et al. 2012
exon 7	c.653_654delCAinsTT	p.A218V	missense	Uematsu et al. 2007
**exon 7**	**c.659G>A**	**p.G220E**	**missense**	**#55/ This study**
**exon 7**	**c.671C>T**	**p.P224L**	**missense**	**#78/ This study**
**exon 7**	**c.710G>A**	**p.G237D**	**missense**	**#92/ This study**
exon 8	c.797A>T^e^	p.H266L^e^	missense	Stadler et al. 2006
exon 8	c.803G>C (r.785_803del)	p.R268T (p.G262_R268delfs*5)	missense/splice	#21, 52, 64/ Holzinger et al. 2001, Dantas et al. 2005
exon 9	c.838G>T	p.D280Y	missense	Uematsu et al. 2007
exon 9	c.845A>G	p.H282R	missense	#34/ Dantas et al. 2005
**intron 9**	**c.903+6_903+9delTACG**	**p.?**	**splice/RNA nd**	**#72/ This study**
exon 10	c.929C>G	p.P310R	missense	#42/ Baumgartner et al. 2001
exon 10	c.994C>T	p.R332*	nonsense	Dantas et al. 2005
exon 11	c.1015G>A	p.V339M	missense	#67, 81, 90, 111/ Baumgartner et al. 2001
exon 11	c.1019A>T	p.D340V	missense	Stadler et al. 2006
exon 11	c.1054G>A (r.1054G>A + r.1000_1072delins r.999+858_r.999+922)	p.G352R + (p.V334_G358delins KFFMKYFLRLDLNSYNSTWQH)	missense/splice (skip exon 11, insert 64 bp from intron 10)	Dantas et al. 2005
exon 11	c.1054_1055delGG	p.G352Rfs*27^f^	deletion/frameshift	Uematsu et al. 2007
exon 11	c.1065A>T	p.L355F	missense	Nguyen et al. 2011
**intron 11**	**c.1073-12C>G (r.1073_1216del+ r.1073insr.1073-48_r.1073-1)**	2 transkripts: (p.G358Vfs*6+p. G358Afs*12)	**splice/2 transkripts: exon 12 and 13 skipping, insertion of 48 bp from intron 11**	**#100/ This study**
exon 12	c.1123G>T	p.V375F	missense	#39/ Dantas et al. 2005
**intron 12**	**c.1149+1G>T**	**p.?**	**splice**	**#136 / This study**
**intron12**	**c.1149+5G>C**	**p.?**	**splice**	**#92/ This study**
exon 13	c.1208A>C	p.N403T	missense	Stadler et al. 2006
**exon 14**	**c.1300G>C**	**p.V434L**	**missense**	**#126/ This study**
exon 14	c.1309A>G(r.1309A>G+ r.1310_1373del64)	2 transkripts: (p.I437V+p.I437Tfs*15)	missense/splice (cryptic splice donor resulting in deletion of the last 64 bp of exon 14)	#111/ Baumgartner et al. 2001
exon 14	c.1367C>T	p.A456V	missense	#37/ Dantas et al. 2005
**exon 15**	**c.1423G>A**	**p.G475R**	**missense**	**#125/ This study**
**exon 15**	**c.1423G>C**	**p.G475R**	**missense**	**#127/ This study**
exon 15	c.1430A>G	p.Q477R	missense	Nguyen et al. 2011
exon 15	c.1465C>T	p.Q489*	nonsense	Stadler et al. 2006
exon 16	c.1549G>A	p.G517R	missense	Nguyen et al. 2011
exon 16	c.1559A>C	p.Y520S	missense	Nguyen et al. 2011
exon 16	c.1567A>G	p.S523G	missense	#56, 69/ Morscher et al. 2011
intron 16	c.1574+1G>A	p.F497Gfs*4^g^	splice, exon 16 skipping	#29, 30/ Dantas et al. 2005
exon 17	c.1624_1625dupGG^h^	p.L543Vfs*11	insertion/frameshift	Uematsu et al. 2007
exon 17	c.1663A>G	p.K555E	missense	Stadler et al. 2006
exon 17	c.1690T>C	p.X564QLE	add 3 aa at C-terminus	#26/ Dantas et al. 2005

(NG_008882.1 [GenBank at the NCBI] was used as reference sequence. Consensus nomenclature according to approved guidelines ( http://www.hgvs.org/mutnomen/)).

nd not detectable

^a^ This mutation has been published as p.L81Lfs*7 in the original paper, nomenclature has been adapted to new approved guidelines.

^b^ For this mutation "MCCB exon 3 skipping, frameshift after residue 66" has been published in the original paper, nomenclature has been adapted to new approved guidelines.

^c^ This mutation has been published as p.G94_S127del in the original paper, nomenclature has been adapted to new approved guidelines.

^d^ This mutation has been published as In5ac-1G→A in the original paper, nomenclature has been adapted to new approved guidelines.

^e^ This mutation has been published as c.979A>T, p.H266L in exon 8 in the original paper. However, c.979A>T would predict an Arginine to Tryptophan change in position 327 (p.R327W). The Histidine to Leucine change in position 266 (p.H266L) could be caused by c.797A>T, therefore, a typing error cannot be excluded.

^f^ This mutation has been published as p.G352RfsX26 in the original paper, nomenclature has been adapted to new approved guidelines.

^g^ This mutation has been published as p.F497_V526>GfsX4 in the original paper, nomenclature has been adapted to new approved guidelines.

^h^ This mutation has been published as c.1625_1626insGG in the original paper, nomenclature has been adapted to new approved guidelines.

### Expression studies and Western blot analysis

The functional consequences of three *MCCC1* (p.E288G, p.G379D, p.I434M) and 8 *MCCC2* (p.S39F, p.G118del, p.Y146N, p.H282R, p.V434L, p.A456V, p.G475R and p.S523G) mutations were investigated by expression studies (Table 7). The *MCCC1* p.E288G and p.G379D mutations showed no residual activity while the p.I434M mutant allele yielded on average 46% of MCC1 wildtype activity.

**Table 7:** **Expression of *MCCC1 *and *MCCC2 *wildtype and mutant alleles**

**Allele**	PCC and MCC activities (pmol/min/mg protein)*					
	**Experiment 1**			**Experiment 2**		
	**PCC**	**MCC**	%**	**PCC**	**MCC**	%**
MCCC1-wildtype	311	193	100	331	157	100
vector only	335	0	0	377	0.3	0.2
MCCC1-p.E288G	283	0	0	352	1.8	1.1
MCCC1-p.G379D	293	0	0	326	0	0
MCCC1-p.I434M	330	87.6	45.4	324	74.5	47.4

MCCC2-wildtype	377	75.4	100	341	49.4	100
vector only	364	0.3	0.4	346	0	0
MCCC2-p.S39F	398	29.6	39.3	368	24.8	50.2
MCCC2-p.G118del	366	10.8	14.3	342	3.2	6.5
MCCC2-p.Y146N	341	58.7	77.9	372	44.1	89.3
MCCC2-p.H282R	301	9.6	12.7	339	2.3	4.7
MCCC2-p.A456V	265	2.0	2.7	340	0.1	0.2
MCCC2-p.S523G	313	56.1	74.4	344	30.9	62.6

MCCC2-wildtype	335	76.9	100	446	43.5	100
vector only	371	0	0	540	0.5	1.1
MCCC2-p.V434L	295	56.6	73.7	449	33.5	77.0
MCCC2-p.G475R	290	33.7	43.8	414	21.1	48.5

Transient expression was performed in transformed MCCC1 and MCCC2 deficient fibroblasts followed by the assay of propionyl-CoA carboxylase (PCC) and 3-methylcrotonyl-CoA carboxylase (MCC) activities. Transfection with an empty vector (vector only) was used as negative control.

*activities are the mean of duplicate determination.

** % of MCC activity of simultaneously expressed wildtype allele.

**Figure 2:**
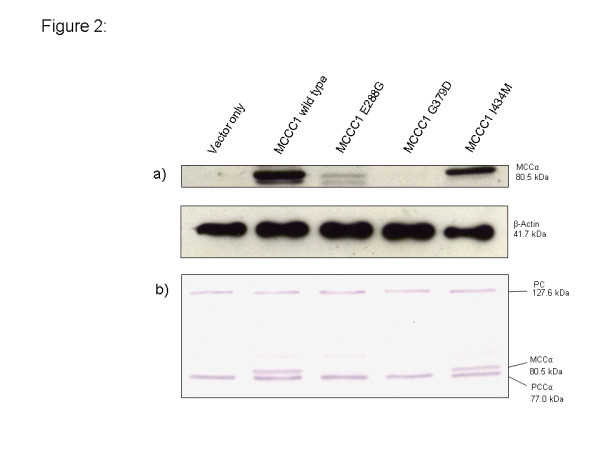
**Western blot analysis of expressed *MCCC1 *wildtype and mutant proteins.** Constructs with *MCCC1* wildtype and 3 mutant cDNAs in pTracer vector were transfected into MCCC1 deficient reference cell lines by electroporation and harvested 48 hours later for Western blot analysis. 50 μg of protein were used per lane. The MCCC1 subunit was visualized by **a)** immunostaining using β-actin (4 μg) as control, or by **b)** colorimetric reaction after coupling with avidin-alkaline phosphatase. Transfection with an empty vector (vector only) was used as a negative control. For further details see “Methods”.

**Figure 3:**
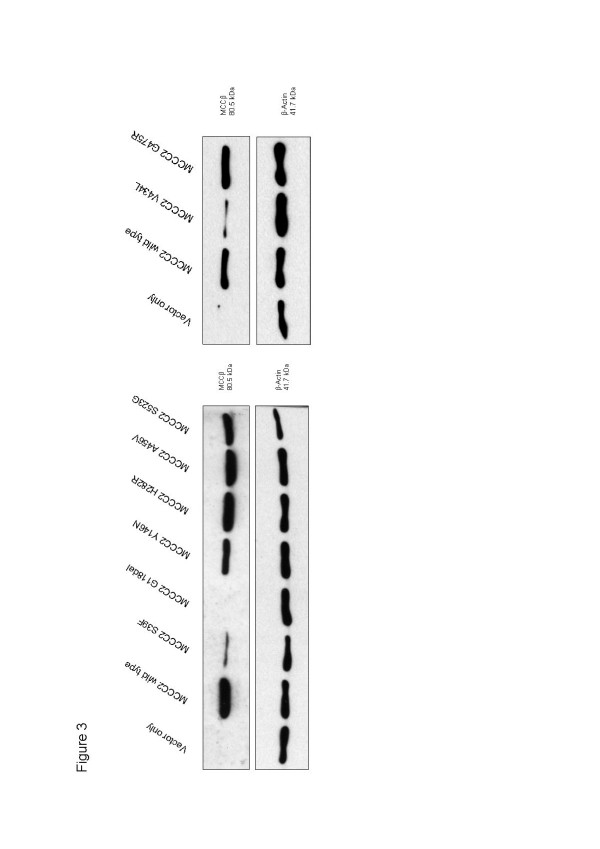
**Western blot analysis of expressed *MCCC2 *wildtype and mutant proteins.** Constructs with *MCCC2* wildtype and 8 mutant cDNAs in pTracer vector were transfected into MCCC2 deficient reference cell lines by electroporation and harvested 48 hours later for Western blot analysis. 50 μg of protein were used per lane. The MCCC2 subunit was visualized by immunostaining using β-actin (6 μg) as control. Transfection with an empty vector (vector only) was used as a negative control. For further details see “Methods”.

Only one of the *MCCC2* mutations, p.A456V, showed virtually no enzyme activity whereas seven mutations were found to have residual activity ranging on average from 9 to 84% of MCC2 wildtype activity. Western blot analysis of the expressed proteins revealed virtually wildtype levels for the *MCCC1* p.I434M protein while the levels of *MCCC1* p.E288G and *MCCC1* p.G379D proteins were severely reduced or not detectable (Figure 2). Five of the 8 expressed *MCCC2* proteins (p.Y146N, p.H282R, p.A456V, p.G475R and p.S523G) were detected at normal or only slightly reduced levels, while the p.S39F and p.V434L protein levels were severely reduced and no protein was detectable after transfection with the p.G118del construct (Figure 3).

## Discussion

This study summarizes clinical, biochemical, enzymatic and mutation data on 88 MCC deficient individuals and summarizes all *MCCC1* and *MCCC2* mutations described so far.

### Clinical phenotype

MCC deficiency has been described as a genetic condition with low clinical penetrance [6]. Results of a comparative analysis of case reports with NBS data reported by Stadler and co-workers [6] suggest that less than 10% of affected individuals ever develop minor symptoms and only less than 1 to 2% might have a risk for severe adverse outcome. In their study all 14 individuals diagnosed by NBS remained asymptomatic during a follow-up period of 1.75 to 6.5 years. In contrast, in our study only 69% of the 36 subjects identified by NBS with a follow-up of at least 3 years have stayed completely asymptomatic while the remainder (31% =11) developed clinical symptoms including various neurologic symptoms as well as at least one acute metabolic decompensation in 5 children. This indicates that early diagnosis with concomitant early initiation of therapy and counselling of parents cannot prevent a clinical manifestation in all cases. However, our data have to be interpreted with caution, since the source of patients being those sent to a diagnostic referral laboratory may lead to a selection bias in favour of symptomatic individuals since it can be assumed that samples for confirmatory diagnosis are more likely to be obtained from symptomatic subjects than from asymptomatic individuals. Additionally, the clinical phenotype of MCC deficiency is still not well-defined.

In our study population the most common clinical features were acute episodes of metabolic acidosis with vomiting, hypoglycemia and acidosis, muscular symptoms such as muscular hypotonia, muscle weakness and muscle pain and neurological abnormalities including developmental delay and seizures as well as attention deficit hyperactivity disorders (Tables 1, 2, 3 and 4). The acute metabolic crises reported in 12 patients are likely to be caused by the underlying metabolic defect. However, the causative attribution of all other clinical signs, especially of unspecific chronic neurologic symptoms such as mental retardation, attention deficit disorders and fatigue to MCC deficiency remains questionable. It could be speculated that these symptoms might as well be caused by undiagnosed genetic defects other than MCC deficiency. Such an additional genetic disorder –though unprobable- would be more likely in individuals that are the product of a consanguineous union; thus a higher share of symptomatic individuals would be expected in this subgroup when compared to subjects whose parents are reported not to be consanguineous. However, no significant difference in the clinical manifestation rate between the two subgroups could be shown (41% symptomatic patients in both subgroups, 12 of 29 individuals with reported parental consanguinity versus 18 of 44 subjects with parents reported not to be consanguineous).

Of the three lethal cases in our study cohort, two could be attributed to a severe metabolic decompensation due to MCC deficiency. The sudden cardiac death of a 9 year old girl was shown to be caused by catecholaminergic polymorphic ventricular tachycardia with mutations in the *RyR2* gene. Thus, in the cohort of 80 individuals of whom clinical information was available, lethality that may be associated with MCC deficiency was 2.5% (2/80).

Altogether, though the share of individuals with clinical symptoms was higher in our study population, our data underline the observation of Stadler and co-workers [6] that compared to other organic acidemias, individuals with MCC deficiency appear to have a significantly higher tolerance toward metabolic stress; even complete absence of MCC activity seems to cause clinical manifestations only in association with environmental triggering factors in a rather small subgroup of individuals. Dietetic treatment is usually not required. However, considering the frequency of carnitine deficiency in our study population, regular monitoring of free carnitine concentrations and – if necessary - oral carnitine substitution seems to be warranted. Also, an emergency regimen during intercurrent illness may be advisable.

### Biochemical phenotype/MCC activity

As shown in Tables 1, 2, 3 and 4 the vast majority of individuals displayed a typical biochemical phenotype with accumulation of metabolites characteristic for MCC deficiency in both blood and urine at the time of diagnosis. Mild elevations of one or more metabolites were the exception and were not more common in asymptomatic individuals. A completely unremarkable urine organic acid pattern was detected in only one woman. However, the concentration of C5OH in her blood was clearly elevated at the same time.

The phenomenon that mutations may cause a clear biochemical phenotype in otherwise asymptomatic individuals is well known from other inborn errors of metabolism implemented in expanded NBS such as isovaleric acidemia [42] or medium-chain acyl-CoA dehydrogenase deficiency [43]. From the data available in this study we were able to confirm earlier observations that in MCC deficiency there is no apparent association between the biochemical and the clinical phenotype and that a mild biochemical phenotype does not seem to be a predictor of a mild clinical expression [2,6,11]. Furthermore, we have recently shown that also individuals who are carriers of a single mutation at the *MCCC1* locus and have residual MCC activity greater than 20% of control may present with a mild biochemical phenotype characteristic for MCC deficiency [12].

In all but two individuals MCC activity in fibroblasts was severely reduced combined with normal activity of PCC. As for the biochemical phenotype, we were not able to demonstrate a correlation between the residual enzyme activity and the clinical phenotype. When individuals with a less severe deficiency of MCC as indicated by a PCC/MCC ratio of < 50 were compared to a subgroup with a more severe deficiency and a ratio of > 50, the manifestation of clinical symptoms was not significantly more common in the latter group (40% (6/15) versus 47% (22/47), respectively).

### Molecular heterogeneity

Molecular genetic analysis of 83 subjects enrolled in the current study revealed a total of 31 new *MCCC1* (n = 15) and *MCCC2* (n = 16) mutations considered to be causative of MCC deficiency. This brings up the total of mutations published to date to 64 for *MCCC1* and 68 for *MCCC2* (Tables 5 and 6) [2-12].

In our study cohort *MCCC2* mutations were 1.7 times more common than *MCCC1* mutations (63% versus 37%, respectively).

Tables 5 and 6 illustrate a broad genetic heterogeneity at both the *MCCC1* and *MCCC2* locus. Mutations are distributed along almost the entire coding regions of both genes with the exception of exon 2 of both the *MCCC1* and *MCCC2* gene which do not host any mutations.

The majority of mutations are private. Notably, in our cohort of 78 families only 5 *MCCC1* and 10 *MCCC2* mutations have been found in more than one family. The most common mutation was the p.R385S mutation in *MCCC1* which was found in 8 individuals and 9 alleles. This missense mutation has been shown to have a dominant negative effect in the presence of the wild type allele and may lead to biochemical and clinical abnormalities in heterozygous individuals [44]. However, in agreement with earlier reports p.R385S appears not to be a predictor of a particular phenotype and has been found in severely affected patients as well as in asymptomatic individuals (this study, [2,3,44].

Another recurring mutation was c.1526delG (p.C509Sfs*14) which was the only mutation found in all 3 individuals from the Faroe Islands suggesting that this is a founder mutation.

### Novel mutations

Among the novel mutations identified in this study (shown in bold in Tables 5 and 6) the most common were missense mutations (n = 18). Frameshift (n = 5) and splice site mutations (n = 5) were more frequent than nonsense mutations (n = 2) and small deletions (of a single amino acid) (n = 1).

 We assume deleterious functional consequences for the frameshift mutations *MCCC1* c.658_662delTCAGA (p.S220Tfs*15), c.1193_1194delTG (p.V398Gfs*19), c.1820delG (p.S607Ifs*5), c.2009_2043del35 (p.A670Dfs*34), c.2123dupA (p.H708Qfs*8)], the splice site mutations *MCCC1* c.639 + 2 T > A (p.S164Rfs*3); *MCCC2* c.903 + 6_903 + 9delTACG (p.?), c.1073-12 C > G (p.G358Vfs*6 + p.G358Afs*12), c.1149 + 1 G > T (p.?), c.1149 + 5 G > C (p.?)] and the nonsense mutations *MCCC1* c.1114 C > T (p.Q372*), c.1882 G > T (p.E628*)] because they result in truncated proteins lacking functionally important domains such as the BC (in *MCCC1*) or CT (in *MCCC2*) domains [2,45].

The *MCCC2* splice site mutation c.1073-12 C > G is interesting since sequence analysis of cDNA of patient #107 revealed two overlapping sequences. One transcript showed exon 12 and 13 skipping, while the other transcript contained an inframe insertion of a 48 bp sequence from intron 11. No wildtype transcript could be detected. It is conceivable that the c.1073-12 C > G mutation creates a cryptic splice site resulting in partial skipping of exons 12 and 13 and in the partial insertion of an additional exon.

**Figure 4:**
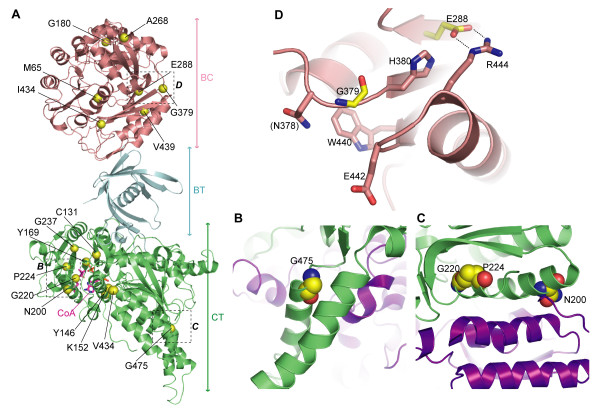
**Mapping of missense mutations onto the P. aeruginosa MCC holoenzyme structure.** (**A**) Mapping of the novel missense mutations (yellow spheres) onto the *P. aeruginosa* MCC holoenzyme structure (PDB code 3U9T), showing that they are clustered in the BC domain of MCCC1 (pink) or the CT domain of MCCC2 (green). (**B**) and (**C**) Mutation sites of Asn200, Gly220, Pro224 and Gly475 are located at the interface between two MCCC2 subunits (coloured green and purple). (**D**) Structural environment of the MCCC1 Glu288 and Gly379 mutation sites. All the residues shown in sticks are conserved between the human and *P. aeruginosa* enzymes. The Glu288-Arg444 ionic interaction is indicated by dashed lines.

Among the novel *MCCC1* missense mutations all variants [p.M65L, p.G180V, p.A268D, p.E288G, p.G379D, p.I434M, p.V439M] change residues within the BC domain, while all novel *MCCC2* missense mutations [p.C131F, p.Y146N, p.K152T, p.Y169D, p.I200N, p.G220E, p.P224L, p.G237D, p.V434L, p.G475R, p.G475R] affect the CT domain of the MCCC2 protein. During the final stage of this manuscript preparation, the crystal structure of *P. aeruginosa* MCC holoenzyme, with >50% sequence identity to the human counterpart (Additional file 1: Figure S1 Additional file 2: Figure S2), was reported [46]. The structure reveals a markedly different holoenzyme architecture compared to other biotin-dependent enzymes, hence providing an unprecedented opportunity to understand MCCC1/2 missense mutations in the protein context. We rationalize that the reported variants are very likely to affect protein function for the following reasons: 1) No other alterations in the *MCCC1* and *MCCC2* gene have been found despite sequencing of the complete coding regions of both genes in all individuals carrying one of those mutations; 2) Amino acid sequence alignments revealed that all but one *MCCC2* c.599 T > A (p.I200N)] of the nucleotide changes affect highly conserved residues across species (PolyPhen2, http:////genetics.bwh.harvard.edu/pph/), and many are also conserved among other biotin-dependent enzymes (Additional file 1: Figure S1, Additional file 2: Figure S2); 3) A majority of the mutations are mapped onto the catalytic core of the MCCC1 BC domain and the substrate binding region of the MCCC2 CT domain (yellow spheres in Figure 4A), suggesting their functional importance; 4) Most of the missense mutations alter the physicochemical properties of the amino acid position (e.g. replacing small with bulky, unipolar with polar, or uncharged with charged residues) and hence are likely to affect the local molecular environment; 5) None of these 19 variants is present in the 1000 Genomes Project dataset ( http:////www.1000genomes.org/home); 6) It is of note that some *MCCC1/2* missense mutations, affecting residues at the inter-subunit interface (Figure 4B and C), may affect not only the corresponding domain fold but also disrupt the assembly of the α and β subunits into the functional hetero-dodecamer.

### Expression data

So far, functional consequences of only 4 MCCC1 and 8 MCCC2 mutant alleles have been proven by expression in a mammalian expression system [2,11]. In this study we expressed 3 further *MCCC1* and 8 *MCCC2* mutations (Table 7) including those expected to show residual enzyme activity based on studies in fibroblasts. Expression of two of the 3 *MCCC1* [p.E288G and p.G379D] and one of the 8 *MCCC2* [p.G118del] mutants resulted in severely reduced MCC activity and protein levels on Western blot analysis confirming deleterious consequences of these mutations on enzyme function and protein stability. Glu288 and Gly379 in MCCC1 are highly conserved residues in the BC domains among biotin-dependent enzymes (Additional file 1: Figure S1). In the *P. aeruginosa* MCC holoenzyme structure, Glu288 forms an ionic pair interaction (Glu288-Arg444) to hold two secondary structure elements together, as observed in the homologous structures of ACC1 (Glu427-Arg604; PDB code 2YL2), ACC2 (Glu533-Arg710; 3GLK) and PCCα (Glu302-Arg459; 3N6R). Gly379 is expected to be at an invariant position at the beginning of a nearby β-strand, directly facing the aforementioned arginine residue. Mutations at Glu288 and Gly379 therefore may disrupt the packing of these secondary structure elements (Figure 4D).

Deleterious effects of two further *MCCC2* mutant alleles [p.H282R and p.A456V] were shown by severely reduced MCC activities of less than 13% of simultaneously expressed wildtype activity. However, in both cases protein levels were normal, and the two affected residues His282 and Ala456 are located at the monomeric surface, indicating that these mutations do not affect the stability of the protein. Expression of all other mutant alleles [*MCCC1* p.I434M, *MCCC2* p.S39F, p.Y146N, p.V434L, p.G475R and p.S523G] yielded considerable levels of residual MCC activity (Table 7). The equivalent of MCCC1 Ile434 in other biotin-dependent carboxylases can be Phe or Leu (Additional file 2: Figure S2), suggesting that substitution to a Met (similar size to Phe and Leu) in the p.I434M mutation may well be tolerated. All mutated residues from the 7 *MCCC2* missense constructs (p.S39F, p.Y146N, p.282R, p.V434L, p.A456V, p.G475R, p.S523G) are at least partially exposed to the surface of the monomer, and not buried within the enzymatic core. Fibroblasts of 4 individuals (No 69, 80, 126 and 127) each homozygous for one of these mutations also showed residual enzyme activity, which was, however, much lower (6.2 – 31% of the median control value) than the activities of the expressed mutant alleles (39% - 77% of simultaneously expressed wildtype activity). Inspite of the high MCC activities after expression Western blot analysis revealed severely reduced protein levels in 2 cases (*MCCC2* p.S39F, p.V434L). Normal levels of protein were expressed by two other mutant alleles (*MCCC2* p.S523G, p.G475R). Even more drastic differences were found between MCC activity of the expressed protein (45%-89%) and that of fibroblasts (3.2% - 7.6%) that are compound heterozygous for the *MCCC1* mutation p.I434M and *MCCC2* mutations p.Y146N and p.S523G (individuals No 56, 66 and 74). Thus, expression studies may not demonstrate/identify the specific functional abnormalities for at least some of the mutant alleles with residual enzyme activity. However, no other mutations were found despite sequencing of the complete coding region of both MCC genes.

### Genotype-phenotype correlations

Our data confirm previous studies reporting no clear genotype-phenotype correlation in MCC deficiency [3,6,11] suggesting that factors other than the genotype at the MCC loci have a major influence on the clinical phenotype [11]. In line with this we identified clinically asymptomatic female adults carrying null mutations in homozygosity and siblings of which one was asymptomatic and the other showed neurologic symptoms compatible with influence of environmental factors on the clinical outcome of affected individuals.

None of the mutations found in asymptomatic individuals with MCC deficiency detected by NBS was prevalent in this group, which is in contrast to other inborn errors of metabolism such as isovaleric acidemia [42] or medium-chain acyl-CoA dehydrogenase deficiency [43]. Consequently, genotyping still appears to be of no help in predicting the clinical outcome of individuals with MCC deficiency.

## Conclusions

Our data confirm that MCC deficiency despite its low penetrance can lead to a severe clinical phenotype resembling classical organic acidurias. However, neither the genotype nor the biochemical phenotype is helpful in predicting which affected individual is at risk of developing clinical symptoms.

## Competing interests

All authors declare that they have no competing interests.

## Authors' contributions

SCG collected and interpreted clinical data, performed expression studies and Western blot experiments and drafted the manuscript; MS, RJM and PB were involved in expression studies and Western blot experiments; TS and BF performed enzyme assays and revised the manuscript; CB carried out the molecular genetic studies; EC, CF, JH, SK, DM, EP, RS, KOS and BW contributed clinical patient data and revised the manuscript; WWY performed sequence alignments and in silico prediction of mutations. He also revised the manuscript; MRB designed the study, was involved in the collection of data, analyzed and interpreted data and drafted/revised the manuscript. All authors read and approved the final manuscript.

## Supplementary Material

Additional file 1 **Figure S1.** Amino acid sequence alignment of human MCCC1. Amino acid sequence alignment of human MCCC1 (hMCCC1, Uniprot Q96RQ3), as well as the structurally characterized *P. aeruginosa* MCCC1 (paMCCC1, Q9I299), *Ruegeria pomeroyi* PCCα (bPCCA, Q5LUF3), human ACC1 (hACC1, Q13085) and ACC2 (hACC2, O00763). Novel MCCC1 missense mutations are asterisked. The electrostatic interaction between Glu288 and Arg444 in MCCC1 is highlighted in green. Click here for file

Additional file 2 **Figure S2.** Amino acid sequence alignment of human MCCC2.Amino acid sequence alignment of human MCCC2 (hMCCC2, Uniprot Q9HCC0), as well as the structurally characterized *P. aeruginosa* MCCC2 (paMCCC2, Q9I297), *Propionibacterium shermanii* methylmalonyl-CoA carboxyltransferase (psMMCC, Q8GBW6), *Streptomyces coelicolor* PCCβ (scPCCB, Q9X4K7) and *Roseobacter denitrificans* PCCα (bPCCA, Q168G2). Novel MCCC2 missense mutations are asterisked. Click here for file
