# Supplementary figures and images for: 3-methylcrotonyl-CoA carboxylase deficiency: Clinical, biochemical, enzymatic and molecular studies in 88 individuals
Source: Orphanet J Rare Dis. 2012 May 29;7:31. doi: 10.1186/1750-1172-7-31 (PMC3495011; doi:10.1186/1750-1172-7-31)

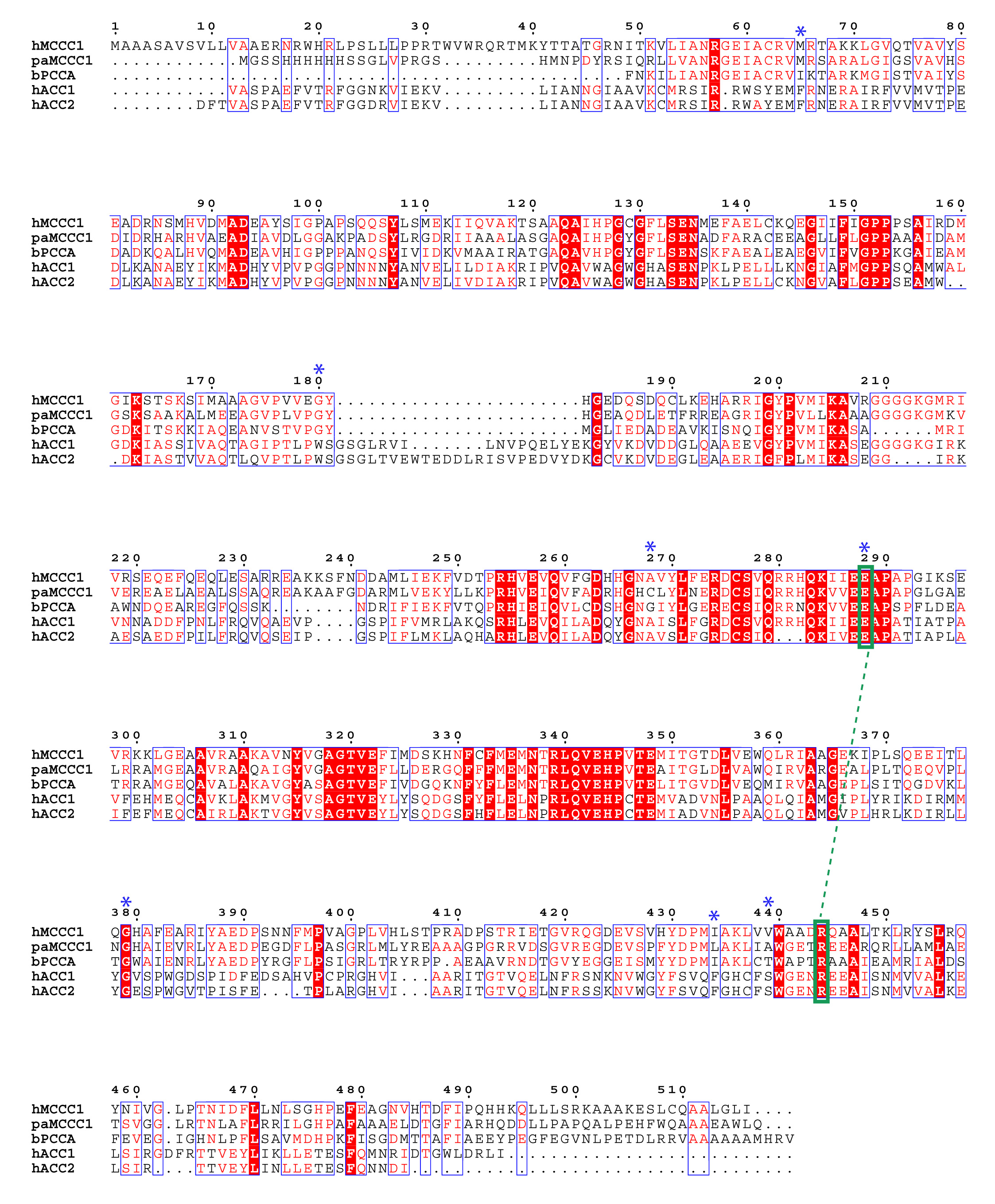

Supplement: Additional file 1 — Figure S1. Amino acid sequence alignment of human MCCC1. Amino acid sequence alignment of human MCCC1 (hMCCC1, Uniprot Q96RQ3), as well as the structurally characterized P. aeruginosa MCCC1 (paMCCC1, Q9I299), Ruegeria pomeroyi PCCα (bPCCA, Q5LUF3), human ACC1 (hACC1, Q13085) and ACC2 (hACC2, O00763). Novel MCCC1 missense mutations are asterisked. The electrostatic interaction between Glu288 and Arg444 in MCCC1 is highlighted in green. [file 1750-1172-7-31-S1.tiff]

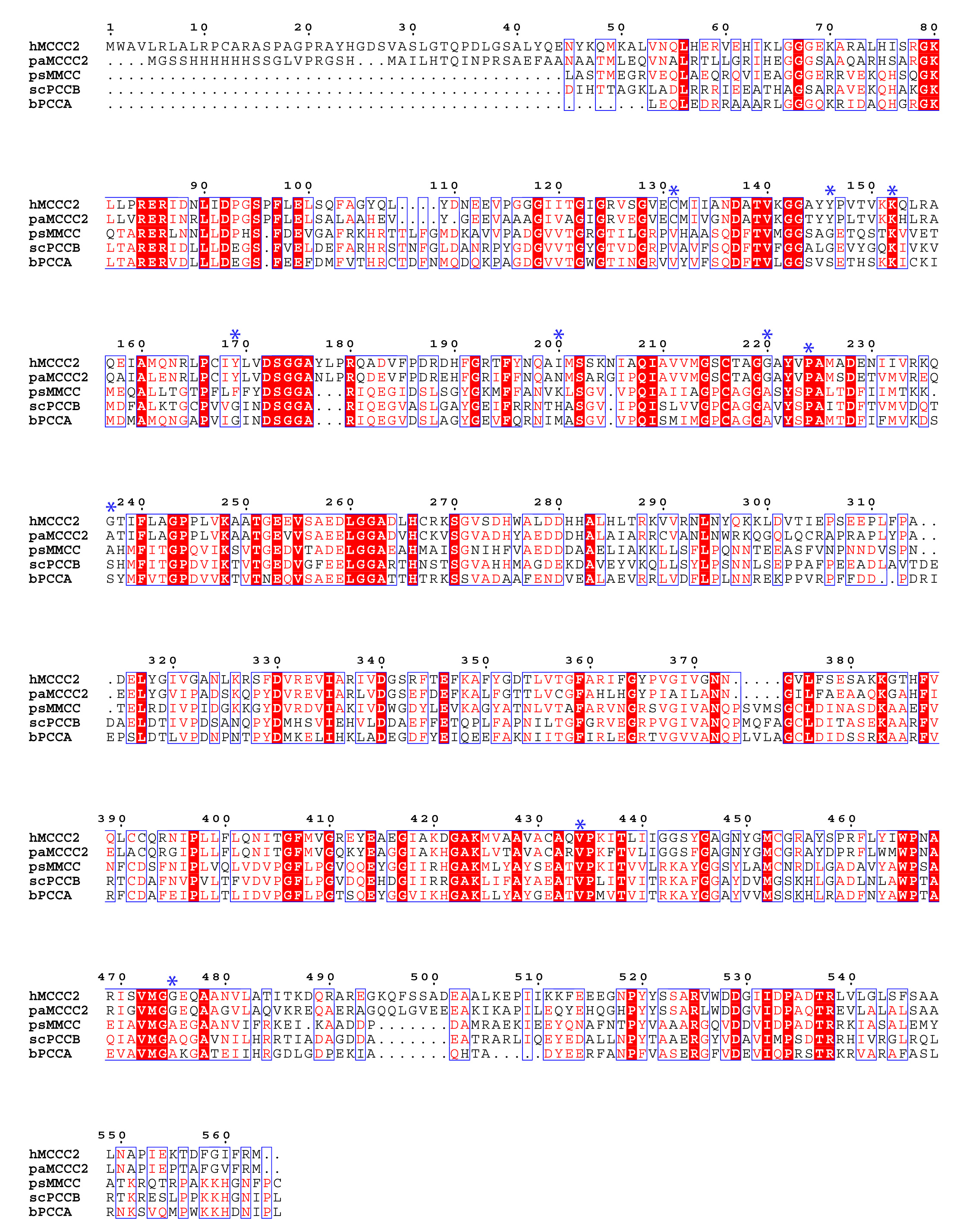

Supplement: Additional file 2 — Figure S2. Amino acid sequence alignment of human MCCC2.Amino acid sequence alignment of human MCCC2 (hMCCC2, Uniprot Q9HCC0), as well as the structurally characterized P. aeruginosa MCCC2 (paMCCC2, Q9I297), Propionibacterium shermanii methylmalonyl-CoA carboxyltransferase (psMMCC, Q8GBW6), Streptomyces coelicolor PCCβ (scPCCB, Q9X4K7) and Roseobacter denitrificans PCCα (bPCCA, Q168G2). Novel MCCC2 missense mutations are asterisked. [file 1750-1172-7-31-S2.tiff]
